# Effects of High-Definition Transcranial Direct Current Stimulation Over the Left Fusiform Face Area on Face View Discrimination Depend on the Individual Baseline Performance

**DOI:** 10.3389/fnins.2021.704880

**Published:** 2021-11-19

**Authors:** Di Wu, Pan Zhang, Na Liu, Kewei Sun, Wei Xiao

**Affiliations:** ^1^Department of Medical Psychology, Air Force Medical University, Xi’an, China; ^2^Department of Psychology, Hebei Normal University, Shijiazhuang, China; ^3^Department of Nursing, Air Force Medical University, Xi’an, China

**Keywords:** high-definition transcranial direct current stimulation (HD-tDCS), fusiform face area (FFA), superior temporal sulcus (STS), face view discrimination, initial performance

## Abstract

A basic human visual function is to identify objects from different viewpoints. Typically, the ability to discriminate face views based on in-depth orientation is necessary in daily life. Early neuroimaging studies have identified the involvement of the left fusiform face area (FFA) and the left superior temporal sulcus (STS) in face view discrimination. However, many studies have documented the important role of the right FFA in face processing. Thus, there remains controversy over whether one specific region or all of them are involved in discriminating face views. Thus, this research examined the influence of high-definition transcranial direct current stimulation (HD-tDCS) over the left FFA, left STS or right FFA on face view discrimination in three experiments. In experiment 1, eighteen subjects performed a face view discrimination task before and immediately, 10 min and 20 min after anodal, cathodal and sham HD-tDCS (20 min, 1.5 mA) over the left FFA in three sessions. Compared with sham stimulation, anodal and cathodal stimulation had no effects that were detected at the group level. However, the analyses at the individual level showed that the baseline performance negatively correlated with the degree of change after anodal tDCS, suggesting a dependence of the change amount on the initial performance. Specifically, tDCS decreased performance in the subjects with better baseline performance but increased performance in those with poorer baseline performance. In experiments 2 and 3, the same experimental protocol was used except that the stimulation site was the left STS or right FFA, respectively. Neither anodal nor cathodal tDCS over the left STS or right FFA influenced face view discrimination in group- or individual-level analyses. These results not only indicated the importance of the left FFA in face view discrimination but also demonstrated that individual initial performance should be taken into consideration in future research and practical applications.

## Introduction

As a kind of visual stimulus or complex object, the face is important to the survival and social communication of various species, including humans. It is a remarkable property for the primate visual system to recognize faces across different viewpoints in invariant views (Axelrod and Yovel, 2012). Humans, for example, can accurately recognize a face despite changes in viewpoint. In real life, we frequently need to recognize a person’s face from various angles. Given that face view processing is a basic ability that people possess, methods that facilitate this kind of face processing could be valuable and attractive due to their importance.

To our knowledge, there could be two important ways to improve or recover functions related to face processing. One is perceptual learning, which refers to a phenomenon in which extensive practice of a perceptual task can boost various perceptual functions (Lu et al., 2011, 2016; Sasaki et al., 2012). Studies have confirmed that face perception can be improved through perception learning (Hussain et al., 2009; Bi et al., 2010; Mcmahon and Leopold, 2012). However, this method is time consuming because it generally needs hundreds or thousands of practice trials over days to weeks to improve. Another method is transcranial direct current stimulation (tDCS), a non-invasive brain stimulation technique that is attracting increasing attention because of its low cost, portability and feasibility (Reinhart et al., 2016; Turski et al., 2017). It has been found that tDCS not only directly boosts perceptual performance (Ding et al., 2016; Reinhart et al., 2016; Wu et al., 2020) but also facilitates perceptual learning, which produces more benefits, such as a reduction in training time, a larger magnitude of improvements and more enduring improvements (Bolognini et al., 2010; Sczesny-Kaiser et al., 2016). More importantly, tDCS contributes to exploring the causality between a certain cortical area and its corresponding functions.

Transcranial direct current stimulation transiently modulates cortical excitability by altering the membrane potential of neurons (Stagg and Nitsche, 2011; Stagg et al., 2011). The technique delivers a mild direct current (DC) between anode and cathode electrodes that are placed on the scalp of a participant. The mild intracerebral DC enters the cortex from the anode and exits the cortex to the cathode. Generally, tDCS effects are bidirectional based on the different directions in the current flow: the anodal electrode increases cortical excitability, and the cathodal electrode decreases excitability (Horvath et al., 2015; Parkin et al., 2015; Woods et al., 2016). The identification of relevant stimulus sites on the scalp is an important question to consider in tDCS studies. There is considerable evidence that humans have specific neural mechanisms for face processing (Kanwisher and Yovel, 2006). It is generally acknowledged that the “core system” for face processing currently includes the fusiform face area (FFA), the inferior occipital gyrus (occipital face area, OFA) and the superior temporal sulcus (STS; Haxby et al., 2000; Gobbini and Haxby, 2007; Fox et al., 2009). Specifically, the FFA is primarily engaged in the perceived identity of the face, whereas the OFA is apparently dedicated to the physical properties of the face stimulus (Rotshtein et al., 2005; Pitcher et al., 2007). STS is related to dynamic aspects of faces, such as their emotional expressions, gazes and viewpoints (Andrews and Ewbank, 2004).

Previous studies may have provided some insights into the neural mechanisms underlying face view discrimination. Studies involving monkeys found face view-selective neuron clustering in the inferior temporal cortex (IT) and STS (Perrett et al., 1985, 1991; De Souza et al., 2005). Early neuroimaging research in humans revealed a greater response in the STS when a face with the same identity was presented from different viewpoints (Andrews and Ewbank, 2004). In contrast, an investigation in terms of perceptual learning of face views demonstrated a close relationship between the left FFA and face view discrimination learning. Specifically, the behavioral learning effects were closely related to improved left FFA stability. Additionally, the pretraining thickness of the left FFA could predict individual behavioral learning effects (Bi et al., 2014). Similarly, an event-related potential (ERP) study also found that the trained face view rather than the untrained view significantly reduced the N170 latency in the left occipital–temporal area (Su et al., 2012). There outcomes in monkeys and humans seem to build close connections between the left cerebral hemisphere and face view discrimination. However, the essential role of the right FFA in face recognition has been extensively documented in literature over the past few decades (Kanwisher et al., 1997). There is a bulk of literature showing a larger response to faces in the right hemisphere than in the left hemisphere (Pinsk et al., 2005; Fang et al., 2007). Thus, the specific cortical region remains controversial.

Recently, tDCS research has found that the results obtained using only the group mean may mask some notable findings, suggesting that analyses of interindividual differences are necessary. For example, previous studies found that neither anodal nor cathodal tDCS over the left dorsolateral prefrontal cortex affected response inhibition measured in a go/no-go task at the group level unless interindividual differences in genetic polymorphisms (Plewnia et al., 2013; Nieratschker et al., 2015) or personality traits (Weidacker et al., 2016) were taken into consideration. Indeed, tDCS data frequently involve high levels of variability across participants, and often, there are some people who show little improvement or even opposite effects after stimulation (López-Alonso et al., 2014; Benwell et al., 2015; Li et al., 2015). Among these interindividual factors that influence tDCS effects, individuals’ initial performance is worthy of attention (Li et al., 2015; Wu et al., 2020). The initial performance can be considered to be related to baseline brain excitability levels that may subsequently determine the stimulation effects on performance (London and Slagter, 2021). In a visual perception study, for example, only anodal tDCS over the primary visual cortex modulated the magnitude of change in contrast sensitivity as a function of individual baseline contrast sensitivity even though both anodal and cathodal tDCS did not influence contrast sensitivity at the group level (Wu et al., 2021). Thus, the current study not only focused on the tDCS effect on face view discrimination at the group level but also further analyzed the individual tDCS effect regarding the correlation between baseline performance and the magnitude of performance change.

Transcranial direct current stimulation is capable of modulating the excitation and inhibition of a certain brain region; therefore, it provides an effective way to tell us about the roles the different cortical areas play in processing face views at different angles. Conventional tDCS typically delivers electrical current in a relatively non-focal manner using a pair of electrodes placed on the scalp (1×1 electrode configuration; Lang et al., 2019). Recently, multielectrode configurations (referred to as high definition; HD) with individual control of current intensity at each electrode allow for unique combinations of electrode locations combined with current optimization algorithms to more focally target brain regions (Dmochowski et al., 2011).

In summary, this research aimed to examine the involvement of the left FFA, left STS and right FFA in face view discrimination using HD-tDCS. In experiment 1, face view discrimination was measured before and after anodal, cathodal and sham tDCS over the left FFA that was counterbalanced across three sessions. In experiments 2 and 3, the target brain region was the left STS or right FFA, respectively. HD-Targets software (Soterix Medical Inc., New York, United States) was employed to define the optimal electrode positions to focally stimulate the left FFA, left STS or right FFA. The modulation of face view discrimination by tDCS was analyzed at the group and individual levels.

## Experiment 1

### Materials and Methods

#### Subjects

Eighteen male subjects (mean age: 20.2 ±0.6 years) had normal or corrected-to-normal vision. Each subject signed a written informed consent form before participating, and they were all naive to the objective of this study. In particular, they were informed that we would apply mild DC on their scalp and that they needed to complete a face-related task four times before and after the application of the DC. None of them had previously participated in tDCS-related experiments. The research received approval from the local Research Ethics Committee and adhered to the principles of the Declaration of Helsinki.

#### Stimuli

**FIGURE 1:**
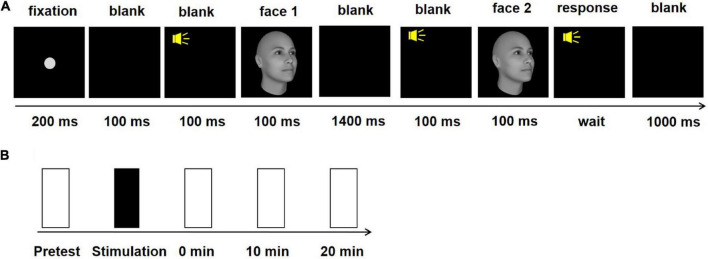
Task and procedure in experiment 1. **(A)** Schematic description of a trial in the 2-AFC face view discrimination task. **(B)** The experimental procedure in one session. The black rectangle indicates anodal, cathodal or sham stimulation. The white rectangles indicate the four test blocks.

FaceGen Modeler 3.5 was used to generate 3D face images at various in-depth rotation angles. The stimuli extended 2° × 2° of the visual angle. One block of face view discrimination included 100 trials and lasted approximately 5 min. Each subject was required to discriminate face views around the in-depth face orientation that was 30° tilted to the right. In a trial (Figure 1A), a 200-ms fixation was presented in the center of the screen followed by a 100-ms blank interval. Two face stimuli (30° and 30 ± θ° face views) were randomly presented in two 200-ms temporal intervals, separated by a 1400-ms blank. A brief tone appeared at the beginning of each interval. During each interval, the face stimuli were displayed in the last 100 ms. The subjects needed to make a two-alternative forced-choice (2-AFC) judgment of the orientation of the second face view relative to the first view (left or right). The step size was 0.2°, with both left and right rotations. They pressed the left button when the second face turned left relative to the first one and pressed the right button otherwise. A brief tone appeared following each response independent of its accuracy, and the next trial began 1000 ms after response.

The θ varied trial by trial and was controlled by an adaptive three-down one-up staircase method to assess subjects’ face view discrimination thresholds that converged to a performance of 79.3% correct. The threshold of face view discrimination was estimated by one block of 100 trials. We recorded a reversal when the direction of the staircase changed from increasing to decreasing θ or vice versa. We deleted the first four (if the total number of reversals was even) or five (if odd) reversals. The threshold for the discrimination of the in-depth orientation of the face view was calculated by averaging the remaining reversals. The starting threshold for each staircase was set near the expected threshold based on pilot testing.

The face images were presented by a computer running MATLAB and PsychToolbox extensions. A gamma-corrected 60×34 cm monitor was used to display the face stimuli, with a spatial resolution of 1920×1080 pixels and a refresh rate of 85 Hz. The subjects viewed the displays binocularly, and their heads were placed on a chin rest to maintain stabilization. The display subtended 6.84° ×3.89° at a 5-m viewing distance. Normal vision was ensured for some subjects through optical correction.

#### Experimental Procedure

The study used a single-blind, sham-controlled within-subject design. All subjects took part in three sessions (anodal, cathodal and sham) with the sequence counterbalanced across subjects. The time interval between each session was at least 48 h to limit potential carryover effects. The threshold of face view discrimination was separately measured four times: before and immediately, 10 min and 20 min after tDCS (Figure 1B). The subjects rested during the stimulation and between the block intervals. After completing all experimental procedures, the subjects were asked to report scalp pain and uncomfortable experiences, and they could not distinguish between active and sham tDCS.

#### High-Definition Transcranial Direct Current Stimulation

**FIGURE 2:**
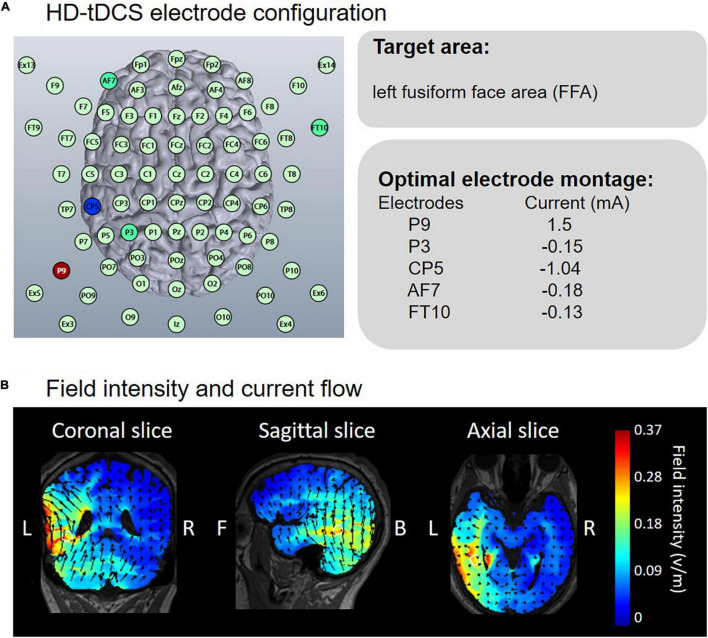
Electrode configuration and simulated electric field in anodal HD-tDCS over the left FFA. **(A)** HD-Targets software defined the optimal electrode montage to focally stimulate the left FFA. **(B)** HD-Explore software modeled the field intensity and current flow.

HD-Targets software, with a finite-element model of a template adult brain to assess the current distribution, was used to confirm the stimulation sites. This software has been proven to have good effectiveness in previous studies (Nikolin et al., 2015; Hartmann et al., 2019; Lang et al., 2019). Electrode positions were selected to generate the highest current focality to the left FFA. Based on the optimized current modeling, electrodes were placed at P9, CP5, P3, AF7, and FT10 (Figure 2A). In the anodal stimulation, P9 served as the anode, delivering an intensity of 1.5 mA DC for 20 min (fade in/out: 30 s). The remaining electrodes receiving the return current were as follows: CP5 = -1.04 mA, P3 = -0.15 mA, AF7 = -0.18 mA and FT10 = -0.13 mA. Conductive gel was used to increase conductivity and reduce impedance. In the cathodal stimulation, the polarity of all electrodes was reversed. Electrode positions in sham conditions were counterbalanced such that the positions corresponding to anodal and cathodal tDCS occurred at equal times. The sham current lasted only 30 s, ramping up at the beginning and down at the end of the 20-min period. Figure 2B depicts the calculated current flow of anodal tDCS using HD-Explore software (Soterix Medical Inc., New York, United States).

#### Data Analyses

SPSS statistical software was used to finish the data analyses. We conducted a two-way ANOVA on the face view discrimination threshold, with groups (anodal, cathodal and sham) and blocks (before and immediately, 10 min and 20 min after) as within-subjects factors to analyze the tDCS effect at the group level. *Post hoc* tests were performed to compare the threshold differences with Bonferroni-corrected *p*.

For the individual analyses, correlation analyses were conducted between an individual’s initial performance and the magnitude of the performance change separately for anodal, cathodal and sham conditions. Furthermore, the analyses of covariance (ANCOVAs) were used to compare the slopes of the two linear models for the anodal/cathodal vs. sham models to exclude the regression effect. The above statistical analyses were performed individually for each of the different blocks.

### Results

#### Group Analyses

**FIGURE 3:**
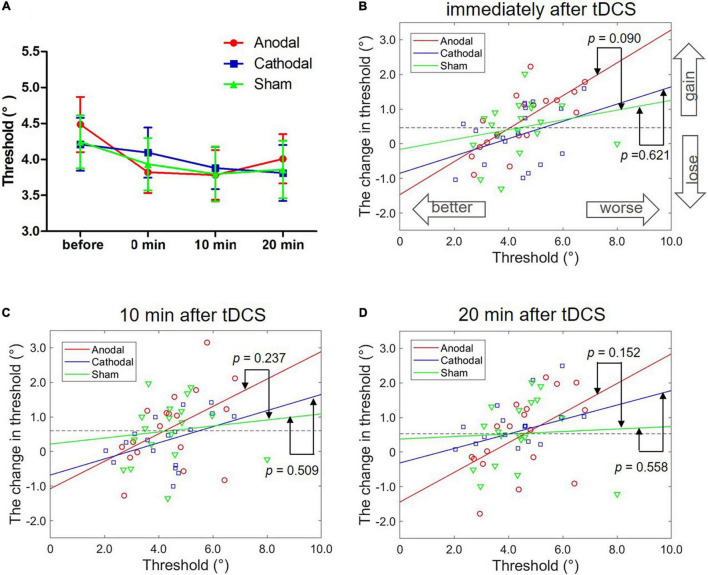
Effects of tDCS over the left FFA on the threshold of face view discrimination. **(A)** The average thresholds are depicted at four times after anodal (red circles), cathodal (blue squares) and sham tDCS (green triangles). Error bars indicate standard errors (SE). **(B–D)** The correlation between the baseline threshold and the change amount in the threshold at different time points. *p-*values represent the significance level between the slopes of two linear models (anodal or cathodal vs. sham).

Three groups and four blocks of two-way ANOVA on the face view discrimination threshold were conducted. Figure 3A shows a significant main effect of block, *F*(3,51) = 5.76, *p* = 0.002, η^2^ = 0.07. Additionally, no significance was found for other effects, *F*s < 1. *Post hoc* tests (with Bonferroni-corrected *p* = 0.017) showed a significantly greater threshold before stimulation than immediately after (*p* < 0.001), 10 min after (*p* < 0.001) and 20 min after (*p* = 0.001) tDCS. There were no significant differences in the thresholds for the three blocks after tDCS (*p*s > 0.1). The possible reason for the significant main effect of block was the practice effect. Theoretically, the practice effect should exist between the first and second tests, and disappear during the following tests. Indeed, we only found a difference in threshold between the first two blocks, and there was no change in the subsequent three blocks of tests. The results indicated no modulation effect of either anodal or cathodal tDCS over the left FFA on the threshold of face view discrimination at the group level.

#### Individual Analyses

We correlated an individual’s initial threshold with the magnitude of the threshold change separately for the anodal, cathodal and sham conditions while controlling for the session order. Regarding the results immediately after tDCS, we found a significant correlation only in anodal tDCS with Bonferroni correction (*r* = 0.70, *p* = 0.002; corrected *p* = 0.017). There were no significant correlations detected in the cathodal (*r* = 0.47, *p* = 0.059) and sham conditions (*r* = 0.29, *p* = 0.263). Nevertheless, the two tests before and after stimulation may have led to a regression effect, a phenomenon in which a variable that is extreme on its first measurement will tend to be closer to the center of the distribution on later measurements. Thus, the significant correlation in anodal stimulation may have resulted from a regression effect instead of the stimulation effect. Here, the results showed a significant correlation only between the initial threshold and the change amount in the anodal condition and not the cathodal and sham conditions, suggesting the existence of a stimulation effect rather than a regression effect.

Comparisons of the slopes in the anodal (or cathodal) vs. sham linear models were conducted to further exclude the regression effect. Specifically, the best-fitting regression lines were estimated with the initial threshold and threshold change through the least square method in the anodal (*r*^2^ = 0.53, *p* < 0.001), cathodal (*r*^2^ = 0.15, *p* > 0.10) and sham (*r*^2^ = 0.05, *p* > 0.10) conditions. ANCOVAs with groups as a fixed factor and the threshold before stimulation as a covariate were performed to compare the slopes of the two models for the anodal/cathodal vs. sham models. Here, ANCOVA was formally equivalent to a moderation analysis in which the initial threshold and groups separately served as continuous and categorial independent variables. The regression effect was supported if the two models (anodal/cathodal vs. sham) were parallel; in contrast, the stimulation effect was supported if the two models were non-parallel. We detected marginally significantly different slopes of the anodal vs. sham linear models, *F*(1,32) = 3.05, *p* = 0.090, η^2^ = 0.07. However, the slope differences between the cathodal and sham linear models were not significant, *F* < 1. These results demonstrated that anodal tDCS over the left FFA can modulate the threshold of face view discrimination in comparison with sham tDCS.

Furthermore, we analyzed the correlation between individual initial thresholds and the magnitude of threshold change 10 min and 20 min after tDCS using the same analytical methods. For the 10 min after the stimulation time point (Figure 3C), no significant partial correlation coefficients were found with Bonferroni correction (corrected *p* = 0.017) in the anodal (*r* = 0.45, *p* = 0.070), cathodal (*r* = 0.35, *p* = 0.176) and sham (*r* = 0.26, *p* = 0.308) conditions. ANCOVAs showed no significant difference in the slope between the anodal and sham models, *F*(1,32) = 1.45, *p* = 0.237, η^2^ = 0.04, or between the cathodal and sham models, *F* < 1. Similarly, for the 20 min after the stimulation time point (Figure 3D), there were no significant partial correlations with Bonferroni correction in the anodal (*r* = 0.50, *p* = 0.042), cathodal (*r* = 0.07, *p* = 0.788) and sham (*r* = 0.31, *p* = 0.232) conditions, and no significant difference in slope between the anodal and sham models, *F*(1,32) = 2.16, *p* = 0.152, η^2^ = 0.06, or between the cathodal and sham models, *F* < 1. Although the results did not reach significance, we still observed a tendency for the slopes with the anodal vs. sham models to be larger than the slopes with the cathodal vs. sham models.

As shown in Figure 3B, data points in the anodal tDCS were distributed on both sides around the dashed line (averaged threshold change in the sham tDCS, 0.46°). For the better initial performers, the threshold changes following anodal tDCS went below the averaged change in sham stimulation and gradually increased with the reduction in the initial threshold. For the poorer initial performers, the threshold changes went above the mean change and improved with increases in the initial threshold. Together, anodal tDCS over the left FFA had diverse effects on face view discrimination dependent on different initial performances, which when combined, offset the group-level effect of tDCS.

## Experiment 2

As mentioned above, the specific cortical region (left FFA, left STS or right FFA) involving face viewing discrimination remains controversial. Experiment 1 provided evidence of the effect of anodal tDCS over the left FFA on face viewing discrimination. Thus, experiment 2 was conducted to investigate the role of the left STS in discrimination.

**FIGURE 4:**
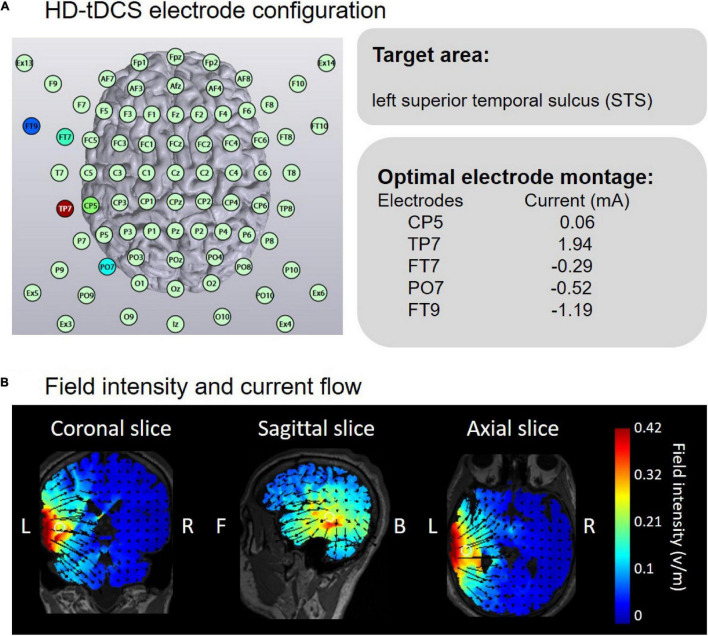
Electrode configuration and simulated electric field in anodal HD-tDCS over the left STS. **(A)** HD-Targets software defined the optimal electrode montage to optimize the focality on the left STS. **(B)** HD-Explore software modeled the field intensity and current flow.

Eighteen male subjects (mean age: 20.5 ± 0.4 years) with normal or corrected-to-normal vision participated in this experiment. All the experimental procedures were the same as in experiment 1. There were two changes in experiment 2: the target brain region was the left STS (Figure 4); and the threshold of face view discrimination was recorded before and immediately after tDCS.

### Results

#### Group Analyses

**FIGURE 5:**
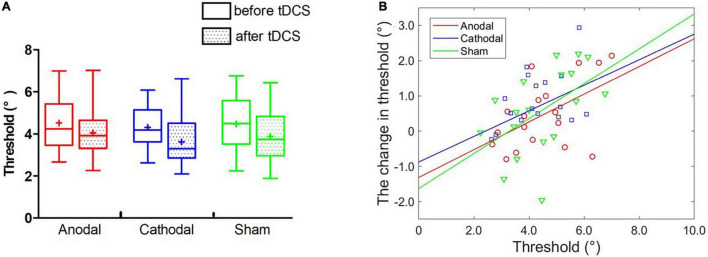
Effect of tDCS over the left STS on the threshold of face view discrimination. **(A)** The average thresholds are depicted before and immediately after tDCS. Error bars indicate standard errors (SE). **(B)** The baseline threshold as a function of the magnitude of threshold change for each type of stimulation.

Similar to experiment 1, two-way ANOVA showed a significant main effect of block, *F*(1,17) = 24.49, *p* < 0.001, η^2^ = 0.15. However, there was no obvious main effect of group, *F*(2,34) = 1.69, *p* = 0.200, η^2^ = 0.03, or interaction effect, *F* < 1 (Figure 5A). The non-significant interaction effect indicated that both anodal and cathodal tDCS over the left STS did not influence the threshold of face view discrimination at the group level in comparison to sham stimulation.

#### Individual Analyses

Partial correlation analyses between the initial thresholds and the change amounts in the threshold were conducted in anodal, cathodal and sham tDCS (Figure 5B). No significant correlations were observed with Bonferroni correction in the anodal (*r* = 0.42, *p* = 0.091), cathodal (*r* = 0.34, *p* = 0.187) and sham (*r* = 0.52, *p* = 0.034) conditions. Furthermore, we estimated the best-fitting lines with the initial threshold and the threshold change amount in the anodal (*r*^2^ = 0.27, *p* < 0.05), cathodal (*r*^2^ = 0.11, *p* > 0.10) and sham (*r*^2^ = 0.26, *p* < 0.05) conditions. ANCOVAs showed no significant differences in the slope of the linear models between the anodal (cathodal) and sham groups, *F*s < 1. The analyses of individual differences further indicated that neither anodal nor cathodal tDCS over the left STS influenced face view discrimination.

## Experiment 3

Experiments 1 and 2 revealed that the left FFA, rather than the left STS, was related to face view discrimination. Some studies have demonstrated a larger response to faces in the right hemisphere than in the left hemisphere (Pinsk et al., 2005; Fang et al., 2007). Thus, the third experiment further investigated how tDCS over the right FFA influences face view discrimination.

Twenty male subjects (mean 19.6 ± 0.6 years) with normal or corrected-to-normal vision took part in this experiment. The objective of this experiment was to further investigate the role of right FFA in face view discrimination using HD-tDCS. Based on the optimized current modeling, electrodes were placed at P10, CP6, P4, AF8, and FT9, which were symmetrical to the electrode positions of left FFA. In the anodal stimulation, the anodal electrode was placed on the P10 (1.5 mA). The remaining electrodes were as follows: CP6 = -1.04 mA, P4 = -0.15 mA, AF8 = -0.18 mA and FT9 = -0.13 mA. In the cathodal stimulation, the polarity of all electrodes was reversed. In addition, all the experimental procedures were the same as in experiment 2.

### Results

#### Group Analyses

**FIGURE 6:**
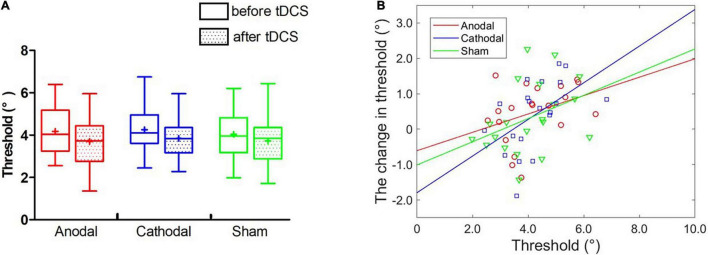
Effect of tDCS over the right FFA on the threshold of face view discrimination. **(A)** The average thresholds are depicted before and immediately after tDCS. Error bars indicate standard errors (SE). **(B)** The baseline threshold as a function of the threshold changes for each condition.

Three groups (anodal, cathodal and sham) and two blocks (pre- and post) of two-way ANOVA showed that the main effect of block was significant, *F*(1,19) = 6.30, *p* = 0.021, η^2^ = 0.11. Additionally, the main effect of group and interaction effect were non-significant, *F*s < 1 (Figure 6A), indicating that both anodal and cathodal tDCS over the right FFA had no effects on face view discrimination threshold at the group level.

Furthermore, we conducted a three-way ANOVA on the face view discrimination threshold, with groups (anodal, cathodal and sham) and blocks (before and immediately after) as within-subjects factors and stimulation sites (left and right FFA) as between-subject factors, to combine the results of Experiment 1 (left FFA) and Experiment 3 (right FFA). The results only showed a significant main effect of block, *F*(1,36) = 15.66, *p* < 0.001, η^2^ = 0.07. No significance was found for other effects, *F*s < 1. There was no significant effect regarding stimulation sites, indicating that the effect of tDCS over the left and right FFAs was not different at the group level.

#### Individual Analyses

Partial correlation analyses showed non-significant relationships between the initial threshold and chance of threshold with Bonferroni correction for anodal (*r* = 0.37, *p* = 0.122), cathodal (*r* = 0.51, *p* = 0.026) and sham (*r* = 0.38, *p* = 0.109) conditions. Furthermore, the best-fitting regression lines were estimated in three tDCS groups (see Figure 6B). ANCOVAs were performed and revealed that the two linear models were parallel independent of anodal vs. sham outcomes, and cathodal vs. sham outcomes, *F*s < 1, excluding the effect of stimulation. These results suggested that anodal and cathodal tDCS over the right FFA did not change face view discrimination at individual level.

Additionally, we compared the slopes of the anodal models between the left and right FFAs. ANCOVAs showed no significant difference in the slopes of the anodal models between the left and right FFAs, *F*(1,34) = 1.23, *p* = 0.276, η^2^ = 0.02.

## Discussion

The current study used HD-tDCS over the left FFA, left STS or right FFA to modulate cortical excitability of these three brain regions and explored whether they were causally related to face view discrimination. Initially, both anodal and cathodal tDCS over the left FFA had no effects at the group level. Interestingly, anodal tDCS, but not cathodal tDCS, over the left FFA modulated the relationship between the individual initial threshold and the magnitude of the threshold change. Specifically, the degree of change after anodal tDCS relied on the initial performance, with poorer (or better) initial performers having a greater gain (or loss). In contrast, neither anodal tDCS nor cathodal tDCS over the left STS or right FFA influenced the threshold of face view discrimination at the group and individual levels. These results indicated that the left FFA seemed to be more susceptible to discriminate face views than the left STS and right FFA.

Interestingly, the effect of tDCS over the left FFA was not found at the group level but at the individual level. As shown in Figure 3B, the data points following anodal tDCS were distributed around the mean level of threshold change following sham tDCS, indicating a convergence effect. In particular, the better initial performers became worse; in contrast, the poorer initial performer improved. Finally, the differential changes averaged together, causing a non-significant change at the group level. Similar outcomes were also found in previous research regarding inhibitory control (Plewnia et al., 2013; Nieratschker et al., 2015; Weidacker et al., 2016), attentional blink (London and Slagter, 2015) and contrast sensitivity (Wu et al., 2021). For example, Wu et al. (2021) did not find modulation of anodal or cathodal stimulation over the primary visual cortex (Oz) on group-level contrast sensitivity compared with sham stimulation. However, initial contrast sensitivity was found to be negatively related to the magnitude of change (more typical at a spatial frequency of 8 c/°) only in the anodal condition, which suggested the involvement of Oz in contrast sensitivity. The convergence effect demonstrates that the various magnitudes of performance change after tDCS depend on the baseline performance. Two studies regarding visual short-term memory also revealed that low initial performers benefited from stimulation, but high performers did not (Tseng et al., 2012; Hsu et al., 2014). Similarly, in a study on attentional blink, participants with a large baseline attentional blink decreased the attentional blink after anodal tDCS, but those with a small baseline attentional blink increased the attentional blink (London and Slagter, 2015). Together, these findings suggest that individual differences in initial performance should be taken into consideration because the group mean results may cover some notable findings.

The convergence effect may have two possible explanations. First, the current intensity and the baseline neural excitability work together to influence tDCS effects. In particular, initial performance may reflect cortical excitability related to the processing efficacy for incoming stimuli (Silvanto et al., 2018). A better initial performance indicates higher excitability; in contrast, a poorer performance signifies lower excitability. Here, the degree of cortical excitability caused by anodal tDCS may be located in the middle position between better and poorer performers. For better performers (high initial excitability), tDCS decreased excitability and then worsened performance. For poorer performers (low initial excitability), tDCS increased excitability and further enhanced performance. Second, the prestimulation cortical excitation/inhibition balance determines the stimulation effects on performance. Specifically, individuals possess various baseline balances between cortical excitation and inhibition within a certain brain area, which influence the stimulation effect based on whether the stimulation moves the balance toward or away from its optimum (London and Slagter, 2021). If a certain brain area already had optimal balance, tDCS would worsen efficiency since the optimal balance is broken. Conversely, if the area has been functioning suboptimally, tDCS would improve its efficiency. Thus, individuals with lower initial performance have suboptimal levels of cortical excitability, and their performance may be improved by tDCS, while individuals with higher initial performance have optimal or supraoptimal cortical excitability, and their performance may be impaired by tDCS. More details regarding the two likely explanations should be investigated in future research.

In an early neuroimaging study, Andrews and Ewbank (2004) found a role of the STS in face view discrimination. Specifically, they showed that a face with the same identity generated greater activation in the STS than faces with different identities when changing the head/gaze direction. Clearly, their study involved face identities using real face images, in addition to face views. In contrast, the current study focused only on the face view using artificial 3D face images. Thus, the above differences between their study and our study may be the reason for the inconsistent findings.

Our findings provide further evidence for hemispheric asymmetry in face processing. Many literatures have documented that the right lateralized responses to faces in the brain were much larger than those in the left hemisphere (Pinsk et al., 2005; Fang et al., 2007), but we still know little about the exact functional difference between these two hemispheres. Meng et al. (2012) proposed different functions in the bilateral cerebral hemispheres. Specifically, the left FFA performs the graded analyses of faces, while the right FFA performs the categorical analyses. Additionally, the left FFA is more susceptible to contextual information than the right FFA. Based on the findings of the current study, we argue that the left FFA is more susceptible to face view discrimination than the right FFA. Our view is consistent with two previous neuroimaging studies (Su et al., 2012; Bi et al., 2014). It’s worth noting that although we confirmed the importance of the left FFA with HD-tDCS, we cannot deny potential contributions from other cortical areas (e.g., right FFA) since tDCS is limited by the low spatial resolution and weak intensity of current to cortex.

The duration of tDCS effects remains controversial. Some early research showed a short-lasting effect of tDCS, such as 7 min (Antal et al., 2001) or 10 min (Antal et al., 2004) after stimulation, which would limit its practical application. To investigate the duration, the threshold of face view discrimination was measured four times: before and immediately, 10 min and 20 min after tDCS. At the group level, neither anodal nor cathodal tDCS influenced the threshold regardless of the duration. Furthermore, the analyses of individual differences immediately after tDCS were significant: the correlation between the initial threshold and the change in threshold was significant after anodal tDCS rather than cathodal and sham tDCS; additionally, the difference in the slope of the linear models between anodal and sham tDCS reached marginal significance. In contrast, the analyses of individual differences at 10 min and 20 min after tDCS were not significant. However, we still found a similar tendency at these three time points. On the one hand, these results verified the reliability of our findings because a similar tendency was found in the three tests at different times. On the other hand, the non-significant results at 10 min and 20 min after tDCS may result from the gradual disappearance of the tDCS effect at these two times.

One potential limitation in this study is the non-specific effects of tDCS on left FFA. In an intact man, the brain is protected from electricity by the skull and by the scalp, both of which normally offer considerable resistance. Thus, the localization of the stimulus on the cortex will always be much less sharp, and the current decays very much. In other words, tDCS is better suited for superficial areas. However, the fusiform gyrus is ventral and medial and the location of the FFA may not be directly accessed. Additionally, it has been found that brain areas are not independent and are especially interconnected. Thus, it is possible that tDCS actually affects the whole network by modulating one part of the network, which may generate unexpected interactions between stimulation sites (Zheng et al., 2011; Krause and Cohen Kadosh, 2014). Given this, there is no direct stimulation of the FFA alone and any effects, if present, cannot be interpreted because of lack of specificity. The current study used HD-tDCS, which has been confirmed to generate more focal current on the target brain region than conventional tDCS with a 1×1 electrode configuration. HD-tDCS is more beneficial by improving the focality of the current and hence potentially limiting the interacting effects among different brain regions. Additionally, the results of this study are consistent with previous fMRI study in which left FFA is related with the face view discrimination (Bi et al., 2014). Thus, it is reasonable to speculate that the left FFA was stimulated in current research.

There were at least two contributions of this study. First, neuroimaging studies have identified the involvement of the left FFA and left STS in face view discrimination. tDCS has advantages in investigating the causal relevance of target brain regions for corresponding cognitive functions. To the best of our knowledge, this research is the first to confirm the role of the left FFA in face discrimination through HD-tDCS, contributing to a deeper understanding of the underlying neuromechanisms of face processing. Second, the previous literature has often used group-level results to validate tDCS effects. However, we did not find a significant influence of anodal tDCS over the left FFA at the group level. In contrast, we found a significantly negative correlation between the initial threshold and the change in threshold, still indicating the role of the left FFA in face view discrimination. These results suggest that the group average results may cover some important findings due to the great variability across individuals. Future research should take individual differences, such as baseline performance, into account.

The current study found that individuals with poorer initial performance showed more improvement following anodal tDCS over the left FFA but not the left STS and right FFA, and further verified the important role of the left FFA in face view discrimination. In future research, individual variability should be taken into account to decrease variability, uncover unclear mechanisms and develop individualized stimulation methods.

## Data Availability Statement

The original contributions presented in the study are included in the article/supplementary material, further inquiries can be directed to the corresponding author.

## Ethics Statement

The studies involving human participants were reviewed and approved by Air Force Medical University. The patients/participants provided their written informed consent to participate in this study.

## Author Contributions

DW completed the experiment and wrote the manuscript. PZ and NL assisted with the experiment and analyzed the data. KS provided technical guidance and site support. WX conceived the idea and provided the financial support. All authors contributed to the article and approved the submitted version.

## Conflict of Interest

The authors declare that the research was conducted in the absence of any commercial or financial relationships that could be construed as a potential conflict of interest.

## Publisher’s Note

All claims expressed in this article are solely those of the authors and do not necessarily represent those of their affiliated organizations, or those of the publisher, the editors and the reviewers. Any product that may be evaluated in this article, or claim that may be made by its manufacturer, is not guaranteed or endorsed by the publisher.
